# PLOD1 Is a Prognostic Biomarker and Mediator of Proliferation and Invasion in Osteosarcoma

**DOI:** 10.1155/2020/3418398

**Published:** 2020-10-19

**Authors:** Haoli Jiang, Wei Guo, Shanyou Yuan, Lixia Song

**Affiliations:** Department of Orthopedics, The Third People's Hospital of Shenzhen, No. 29, Buji Bulan Road, Longgang District, Shenzhen, China

## Abstract

**Objective:**

Osteosarcoma is the most common primary bone tumor and most frequently develops during adolescence. PLOD family was mainly involved in lysyl hydroxylation and rarely investigated in cancers, especially in osteosarcoma. The aim of this study was to investigate the expression pattern and oncogenic role of PLODs in osteosarcoma.

**Methods:**

GEO datasets (GSE16088, GSE33382, and GSE16091) and validation cohort were used to analyze the expression pattern of PLODs in osteosarcoma. Kaplan-Meier survival analysis was used to explore the prognostic role of PLODs in patients with osteosarcoma. RNA interference of KRT19 was performed using small interfering RNA (siRNA) in MG-63 and U-2OS cells. The proliferation was detected using CCK8, clone formation assay, and EdU staining. Migration and invasion were determined using the transwell assay. Western blots and luciferase assays for *β*-catenin-T-cell factor protein/*β*-catenin-lymphoid enhancer factor- (*β*-catenin-TCF/LEF-) driven transcriptional activity.

**Results:**

PLOD1 was upregulated in osteosarcoma tissues compared with control tissues both in public datasets and in in-house cohort. The expression of PLOD1 in osteosarcoma tissues was significantly associated with the status of distance metastasis and Enneking stage, while PLOD2 and PLOD3 expressed no difference between osteosarcoma and benign tissues and showed no correlation with tumor malignancy. Furthermore, Kaplan-Meier survival analysis revealed that patients with a higher level of PLOD1 had worse prognosis than those with a lower level of PLOD1. Downregulation of PLOD1 dramatically inhibited proliferation, migration, and invasion of MG-63 cells and U-2OS cells in vitro. Mechanistically, PLOD1 regulated *β*-catenin signaling pathway in osteosarcoma.

**Conclusion:**

Our results indicated that PLOD1 promoted proliferation, migration, and invasion of osteosarcoma cells. PLOD1 was a novel prognostic marker, as well as a therapeutic target in osteosarcoma.

## 1. Introduction

Osteosarcoma is the most common tumor of the bone in adolescents [1, 2]. The incidence of patients aged 0-24 years was between 2.0% and 7.6% depending on the regions [3]. It was reported that there were about 20% of osteosarcoma patients presenting clinical metastasis to the lungs at the time of initial examination and the 5-year survival rate was less than 20% in these patients [4]. Treatments of osteosarcoma mainly relied on surgery and chemotherapy. However, there were still many patients who suffered recurrence or distant metastasis, especially patients who presented clinical metastasis to the lungs at the time of initial examination [5, 6]. It was reported that the 3-year event-free survival (EFS) and 5-year overall survival of OS patients were 40% and less than 20% in these, respectively [7–9]. The malignant progression of tumors depended on the regulation of cancer-associated molecules, so it is particularly important to find novel markers that are significantly associated with the malignant phenotype and clinical prognosis of osteosarcoma.

New evidences in the field of epigenetics research indicated that osteosarcoma was caused by genetic alteration and genetic accumulation [10, 11]. Procollagen-lysine, 2-oxoglutarate 5-dioxygenase (PLOD) family includes 3 members that are mainly responsible for lysyl hydroxylation, of which mutations have been shown to cause Ehlers-Danlos syndrome as well as Bruck syndrome type 2 [12]. It was reported that PLOD1 and PLOD2 genes specifically function as a telopeptide lysyl hydroxylase, which promoted cross-linking in ECM molecules, which contribute to ECM structural stability and maturation [13]. An increasing number of studies have demonstrated a critical role for PLOD family in the development and progression of cancer. A previous study assessed the expression and prognostic value of PLOD1/2/3 in wild-type and genetically mutated clear cell renal cell carcinoma (ccRCC) patients. Compared with the paired normal kidney tissues, ccRCC tissues expressed notably high PLOD1/2/3 mRNA and protein [14]. Here, high-level PLOD1/2/3 expression was strongly linked to advanced pathological tumor stage and overall survival. Moreover, PLOD1/2/3 were also reported to be associated with several other solid tumors, such as gastrointestinal carcinoma and glioma [15, 16], while the expression and prognostic role of PLODs in osteosarcoma remain to be further illustrated.

In the present study, we used bioinformatics and in-house cohort to analyze the differences in PLOD1/2/3 gene expression between osteosarcoma and normal tissue. In addition, we identified the relationship between PLOD1/2/3 expression and clinicopathological characteristics as well as patients' prognosis. Moreover, the potential gene functional analysis was involved to explore the prospective mechanisms in the pathogenesis of osteosarcoma.

## 2. Methods

### 2.1. Patient Samples

This study was carried out on 56 osteosarcoma tissues that were collected from the Department of Orthopedics of the Third People's Hospital of Shenzhen. There were 19 patients with lung metastasis presented at diagnosis and 30 patients diagnosed as osteosarcoma at advanced Enneking stage. Another 10 patients with benign bone tumor tissues were also collected. None of the patients received any chemo- or radiotherapy before surgery. This study received approval of the Ethics Committee of the Third People's Hospital of Shenzhen. All patients included in this study signed informed consent.

### 2.2. Bioinformatics Analysis

Normalized gene-level RNAseq and clinical data of GSE16088, GSE33382, and GSE16091 were downloaded from the GEO database (https://www.ncbi.nlm.nih. gov/geo/).

The expression of genes between osteosarcoma tissues and normal tissue in GSE16088 and GSE33382 datasets was used. GSE16091 dataset contains substantial information of gene expression profiles and clinical prognosis.

### 2.3. Cell Culture and Transfection with Interfering RNAs

MG-63 and U-2OS cells were purchased from the Cell Bank Type Culture Collection of the Chinese Academy of Sciences (Shanghai, China). Cells were cultured with RPMI 1640 supplemented with 10% fetal bovine serum (Gibco, USA) in a cell culture incubator. siRNAs were all purchased from RIBOBIO (Guangzhou, China). Briefly, cells were trypsinized, counted, and seeded at 1 × 10^5^ cells/well into six-well culture plates, so that the cells were 50%–60% confluence on the day of transfection. siRNA transfection reagent complexes were then added to the antibiotic-free differentiation medium, and myotubes were incubated for 24 h in the medium containing the transfection mixture. Lipofectamine 2000 was used for transfection according to the instructions. Transfected cells were used for subsequent experiments for 48 h after transfection.

### 2.4. Western Blot

Total cellular protein was extracted using RIPA cell lysate. The protein concentration was measured using a bicinchoninic acid assay kit, and an equal amount of 20 *μ*g protein was separated by 8–12% SDS-polyacrylamide gel electrophoresis. The proteins were transferred to PVDF transfer membrane (1 h transfer at constant current of 200 mA). Proteins were transferred to polyvinylidenedifluoride (PVDF) membranes that were next blocked with 5% milk in TBST for 1 h and washed three times in TBST for 5 min. Then, primary antibody incubation (overnight) and secondary antibody incubation for 1 hour were performed, respectively, and finally scanned with an Odyssey scanner. The main antibodies used were as follows: AKT (Proteintech, No. 10176-2-AP), Phospho-ser473-AKT (Proteintech, No. 66444-1-Ig), *β*-catenin (Abcam, ab16051), GAPDH (Bioword, MB001), MMP2 (Bioword, BS72289), and vimentin (Santa Cruz, sc-6260).

### 2.5. RNA Isolation and RT-PCR

Total RNA was extracted from osteosarcoma tissues and cells by using TRIzol reagent (Invitrogen). PrimeScript RT reagent kit with gDNA Eraser (TaKaRa, Tokyo, Japan) was used to prepare cDNA, and SYBR Green II Mixture (TaKaRa) was used for real-time PCR. All operations were performed according to the manufacturer's protocol. The primers used are as follows: PLOD1—forward primer CGCCATGGATCTGTGGACCTGTT and reverse primer CGGGTCCACTTATGGCATCCGAG; PLOD2—forward primer CAGTTCCAGTGCGCTGGATT and reverse primer CCGCTCAGATGGTGGTCTAT; PLOD3—forward primer ATACACTGTCTCGGACGCC and reverse primer TATTGAGATGACGAGAGCCAGC; CTNNB1—forward primer 5′-GCACCACAAGGACATACAGTCA-3′ and reverse primer 5′-CTGCAGACGATGAATGCGAACA-3′; and GAPDH—forward primer 5′-CGAGATCCCTCCAAAATCAA-3′ and reverse primer 5′-TTCACACCCATGACGAACAT-3′.

### 2.6. Cell Proliferation Detection

In order to detect cell viability, 96-well plates (3 × 10^3^ cells per well) were utilized to immediately seed cells or 1 day after transfection. A cell counting kit 8 (CCK8) assay was carried out using a CCK8 assay kit (Boster, China) to evaluate the proliferative activity following the manufacturer's instructions. The MG-63 and U-2OS cells were put in a 96-well plate (5000 cell/well). Cell viability was monitored at 0 h, 24 h, and 48 h. To examine cell viability, 10 *μ*L of CCK8 reagent was added into each well of the 96-well plate. Cell viability was calculated using the following equation: cell viability (%) = OD‐treated cells/OD control cells × 100%. For the clone formation assay, 1000 cells per well were seeded in 6-well plates and cultured for 2 weeks. After 14 days of incubation, they were washed and stained with crystal violet, and colonies containing >50 cells were counted as clonogenic survivors. For cell EdU immunofluorescence staining, MG-63 and U-2OS cells were seeded into 96-well plates and performed using the EdU Kit (RiboBio).

### 2.7. Transwell Assay

20,000 cells were placed on the top of the room with or without Matrigel and cultured with serum-free medium. 600 *μ*L of complete medium was added in the lower room. Cells were cultured for 48 hours in a cell culture incubator. Then, cells were washed with PBS, fixed with 4% paraformaldehyde, and stained with 0.2% crystal violet. Photographs were captured by using an inverted microscope, and six high-power fields were randomly selected for cell counting.

### 2.8. TCF/LEF Luciferase Reporter Assay

For luciferase reporter gene assay, cells were seeded onto 6-well plates at 5 × 10^5^ cells/well and subsequently transfected for 2 days. Cells were transiently cotransfected with the TCF/LEF firefly luciferase construct (100 ng) and the Renilla luciferase vector (10 ng) (Yeasen Biotech Co., Ltd., Shanghai, China). After 24 h, the cells were harvested and analyzed for firefly luciferase and Renilla luciferase activity using the dual luciferase reporter assay kit (Yeasen Biotech Co., Ltd., Shanghai, China) according to the manufacturer's protocol.

### 2.9. Immunofluorescence Staining

In short, the circular glass slices were placed in six-well plates; transfected cell suspensions were added and then cultured in an incubator for 6 h in 5% CO_2_ at 37°C to make cell climbing pieces. Subsequently, the climbing pieces were washed three times with PBS, fixed with 4% paraformaldehyde for 15 min, and washed with PBS for three times. Cells were then permeabilized (PBS + Triton 0.1%) and blocked in 1% BSA for 1 h at room temperature. The primary antibody was diluted in the blocking buffer to incubate cells overnight at 4°C. Alexa Fluor anti-rabbit antibody (Invitrogen, Lot. A-11034) with green fluorescence was used as a secondary antibody and incubated at room temperature avoiding light for 1 h. After being stained with DAPI avoiding light at room temperature for 5 min, the slice was added with an appropriate amount of antifluorescence quencher and observed under a fluorescence microscope.

### 2.10. Gene Function Analysis (GO Enrichment)

Differential gene (DEG) expression analysis between the high and low PLOD1 groups was performed by GEO2R website (https://www.ncbi.nlm.nih.gov/geo/). DEGs were chosen by the criteria  | FC | ≥2 and *P* ≤ 0.05. Metascape is a comprehensive tool for gene annotation and enrichment analysis [17]. To interpret the specific functions and pathways associated with DEGs, genes in selected modules were analyzed using the online bioinformatics database Metascape (http://metascape.org) [17].

### 2.11. Statistical Analysis

Continuous variables were presented by the mean ± standard deviation, and the median was used for categorical variables. A *t*-test was used for comparisons between two groups. One-way ANOVA was used for the analysis of three or more group comparisons, and an LSD *t*-test was used as postanalysis for the comparison between two groups. Box-plot was used to present the levels of PLODs between different groups. Kaplan-Meier survival analysis was performed to explore the relationship between PLODs and prognosis. Univariate and multivariate Cox regression analyses were used to analyze the independent risk factors of clinical outcome. Picture production was performed by GraphPad 8.0. Statistical analyses were conducted using SPSS 21. *P* < 0.05 was defined as a statistical difference.

## 3. Results

### 3.1. PLOD1 Elevated in Osteosarcoma Tissues and Correlated with Tumor Malignancy

**Figure 1:**
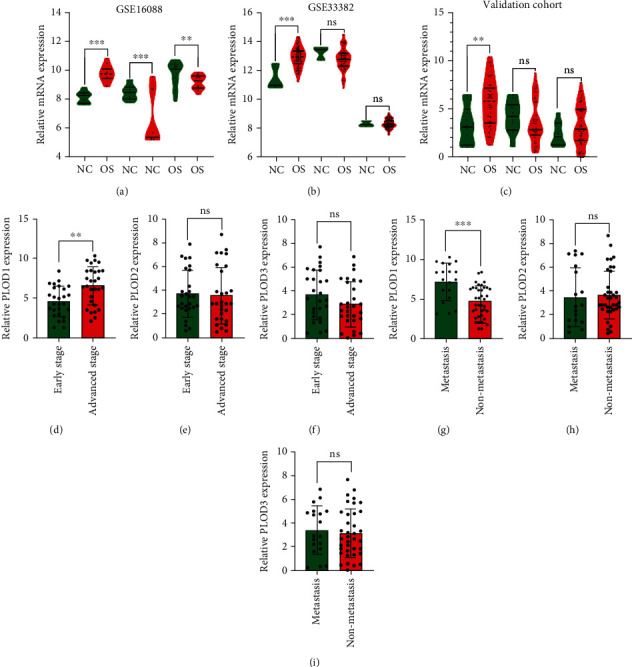
Expression of PLODs in osteosarcoma tissues. (a, b) Relative mRNA expression of PLOD1/2/3 in GSE16088 and GSE33382, respectively. (c) RT-PCR was performed to detect expression of PLODs in 56 osteosarcoma tissues and 10 benign bone tumors in our validation cohort. (d–f) Relative mRNA expression of PLOD1, PLOD2, and PLOD3 in osteosarcoma patients with or without distance metastasis at diagnosis, respectively. (g–i) Relative mRNA expression of PLOD1, PLOD2, and PLOD3 in osteosarcoma patients with different Enneking stages. ^∗∗^*P* < 0.01; ^∗∗∗^*P* < 0.001; ns: no significance.

We used GSE16088 and GSE33382 dataset to investigate the expression of PLODs in different tissues. We found that PLOD1 was upregulated in osteosarcoma tissues when compared with normal bone tissues in GSE16088 and GSE33382 (Figures 1(a) and 1(b)), while the expression pattern of PLOD2 and PLOD3 was not consistent in two public datasets. Osteosarcoma tissues expressed no significant PLOD2/3 than normal bone tissues in GSE33382 (Figure 1(b)). To verify our findings, we performed RT-PCR to detect expression of PLODs in 56 osteosarcoma tissues and 10 benign bone tumors. Similar with our previous results, we found that only PLOD1 was higher in osteosarcoma tissues when compared with benign tumors (Figure 1(c)). Many previous studies have reported molecular mechanisms underlying osteosarcoma that were involved in the progression and metastasis of osteosarcoma [18, 19]. Results of RT-PCR in 56 osteosarcoma tissues revealed that expression of PLOD1 was higher in patients with lung metastasis presented at diagnosis than those without metastasis (Figure 1(d), *P* < 0.01), while PLOD2 and PLOD3 expression presented no significance between two groups (Figures 1(e) and 1(f), all *P* > 0.05). Furthermore, the expression of PLOD1 significantly increased in patients in advanced Enneking stage when compared with those in the early stage (Figure 1(g), *P* < 0.001). However, no significant difference was noted in the expression level of PLOD2/3 between early stage and advanced stage osteosarcoma tissues (Figures 1(h) and 1(i), all *P* > 0.05).

### 3.2. PLOD1 Associated with Prognosis of Osteosarcoma Patients

**Figure 2:**
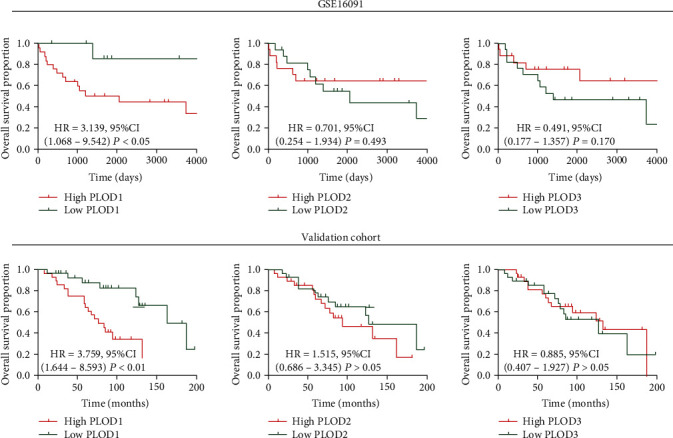
Association between PLODs and clinical prognosis. GSE16091 and validation cohort were used to investigate correlation between PLOD expression and prognosis of osteosarcoma patients. 50% of gene expression was used as a cutoff point in Kaplan-Meier analysis. HR: hazard ratio; CI: confidence interval.

To explore the prognostic role of PLODs, we performed Kaplan-Meier survival analysis. Our results showed that patients with higher expression of PLOD1 had worse prognosis than those with lower expression of PLOD1 in GSE16091, while no such correlation was observed between PLOD2/3 and prognosis. Moreover, results of Cox regression analysis showed that a higher PLOD1 level was an independent risk factor of overall survival in patients with osteosarcoma (Table 1). To further confirm the prognostic role of PLOD1, we used a validation cohort. Consistent with the results of GSE16091, only PLOD1 expression was associated with the prognosis of patients with osteosarcoma (Figure 2).

### 3.3. GO Enrichment Analysis and Pathway Prediction of PLOD1

**Figure 3:**
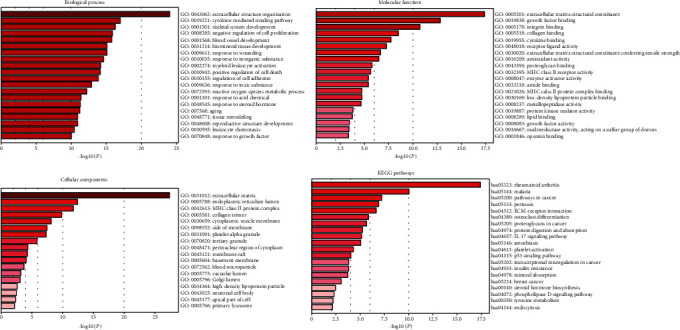
GO enrichment analysis and pathway prediction of PLOD1. GO functional enrichment analysis and KEGG pathway enrichment analysis were performed on the differentially expressed genes (DEGs). Annotations are grouped by biological process, cellular component, and molecular function.

GO functional enrichment analysis and KEGG pathway enrichment analysis were performed on the differentially expressed genes (DEGs) (up- or downregulated). Annotations are grouped by a biological process, cellular component, and molecular function based on the GO annotation information. Bioinformatics analysis of microarray results showed that the identified DEGs were involved in multiple biological processes, such as extracellular structure organization (GO:0043062), cytokine-mediated signaling pathway (GO:0019221), and skeletal system development (GO:0001501). Under “molecular function,” “extracellular matrix structural constituent (GO:0005201),” “growth factor binding (GO:0019838),” and “integrin binding (GO:0005178)” were the most enriched terms (Figure 3). Finally, terms associated with cellular component that were most enriched were membrane related (GO:0031012, GO:0005787, and GO:0042613). For the KEGG pathway enrichment analysis, our results showed that DE genes were significantly enriched in rheumatoid arthritis (hsa05323), malaria (hsa05144), and pathways in cancer (hsa05200) (Figure 3).

### 3.4. PLOD1 Promotes Proliferation of Osteosarcoma Cells

**Figure 4:**
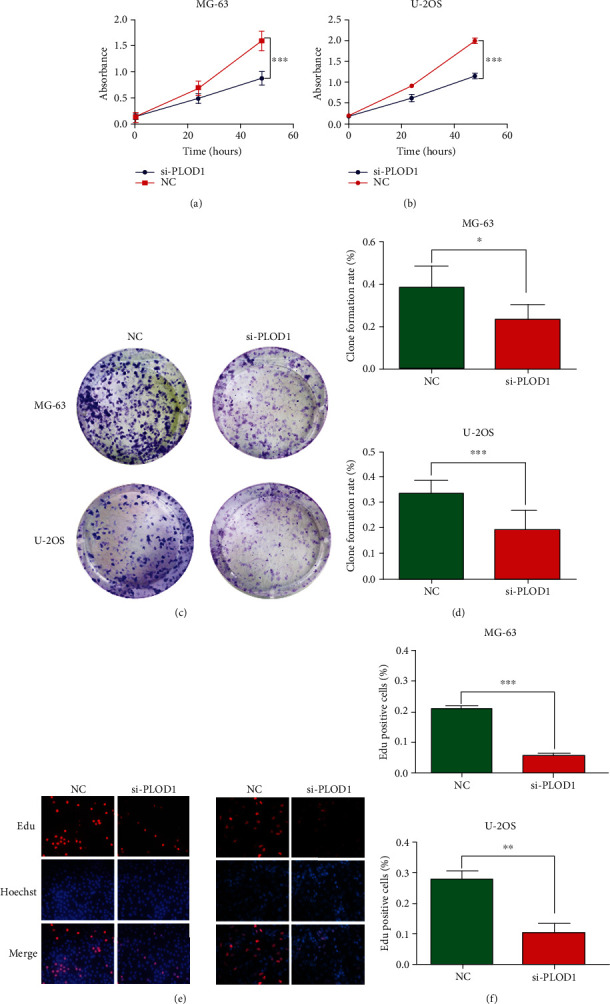
PLOD1 promotes proliferation of osteosarcoma cells. (a, b) CCK-8 was used to detect the effect of PLOD1 on proliferation. Cell viability was monitored at 0, 24, and 48 h. Clone formation (c, d) and Edu (e, f) were employed to measure the ability of proliferation of MG-63 and U-2OS cells after inhibiting PLOD1 expression. ^∗^*P* < 0.05; ^∗∗^*P* < 0.01; ^∗∗∗^*P* < 0.001.

Our above results indicated that PLOD1 was the only gene in the PLOD family, which might function in the malignancy processes of osteosarcoma. To explore the biological function of PLOD1, we performed in vitro experiments using MG-63 and U-2OS cells. First, in the CCK8 assay, we found that PLOD1 knockdown significantly inhibited the proliferation of MG-63 and U-2OS cells (Figures 4(a) and 4(b)). Consistent with CCK8 results, colony formation (Figures 4(c) and 4(d)) and Edu assay (Figures 4(e) and 4(f)) results further demonstrated PLOD1 inhibition on MG-63 and U-2OS cell proliferation.

### 3.5. PLOD1 Enhanced Migration and Invasion of Osteosarcoma Cells

**Figure 5:**
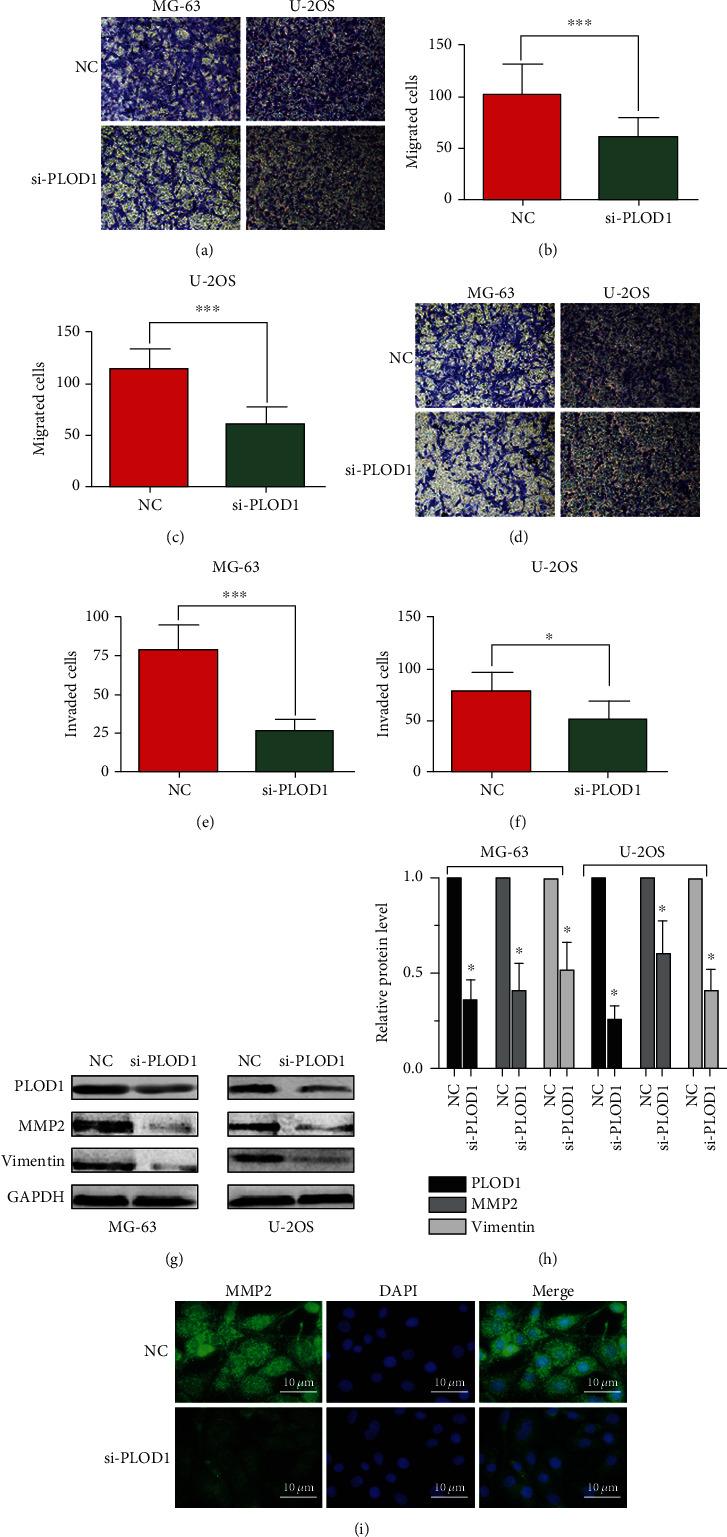
PLOD1 promotes migration and invasion of osteosarcoma cells. Transwell assay was used to detect the effect of PLOD1 on migration (a–c) and invasion (d–f) of MG-63 and U-2OS cells. (g, h) Western blot was performed to detect expression of MMP-2. (i) IF staining of vimentin in U-2OS cells. GAPDH was used as loading control. NC: normal control. Scan bar, 10 *μ*m. ^∗∗∗^*P* < 0.001.

Using transwell assays, we demonstrated that knocking down PLOD1 expression significantly attenuated MG-63 and U-2OS cell migration capacities compared with the controls (Figures 5(a)–5(c)). Also, when the upper chamber was coated with Matrigel, we found that inhibition of PLOD1 reduced the invasion of MG-63 and U-2OS cells (Figures 5(d)–5(f)). In addition, western blot analysis was performed to determine whether the protein levels of invasion-associated markers were also affected by PLOD1. Our results showed that inhibition of PLOD1 in MG-63 and U-2OS dramatically reduced expression of mmp2 and vimentin (Figure 5(g)). To determine vimentin expression after knocking down PLOD1 in osteosarcoma cells, we performed immunofluorescence (IF) staining, and the results also confirmed previous results using WB (Figure 5(f)).

### 3.6. PLOD1 Regulated *β*-Catenin Signal Pathway

**Figure 6:**
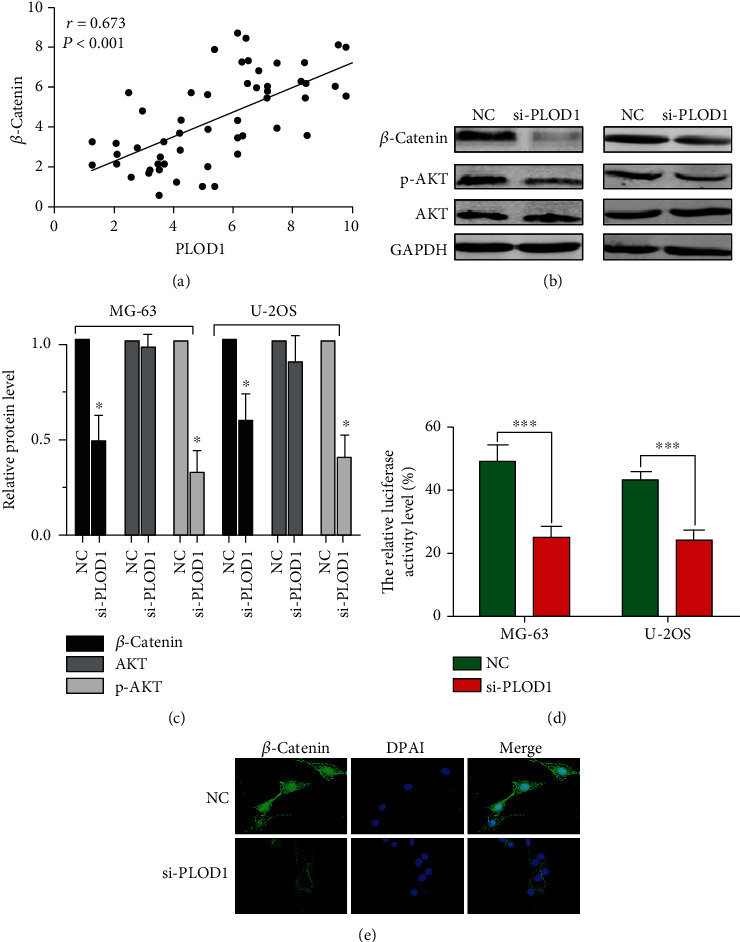
PLOD1 regulated *β*-catenin signal pathway. (a) Correlation between PLOD1 and *β*-catenin mRNA expression. (b, c) Expression of *β*-catenin, p-AKT, and total AKT detected by western blotting. GAPDH was used as loading control. NC: normal control. (d) *β*-Catenin–luciferase levels in total cell lysates, as assayed by measuring luciferase activity. (e) IF staining of *β*-catenin showed the relative expression levels of *β*-catenin proteins in cytosol or nucleus in U-2OS cells. Scan bar, 10 *μ*m. ^∗∗∗^*P* < 0.001.

Wnt/*β*-catenin played a crucial role in regulating the proliferation and invasion of osteosarcoma cells. Using RT0-PCR, we found that expression of PLOD1 significantly correlated with *β*-catenin expression in 56 osteosarcoma tissues (*r* = 0.67 and *P* < 0.01, Figure 6(a)). In this study, we found that inhibition of PLOD1 expression in osteosarcoma cells dramatically reduced the expression of p-AKT and *β*-catenin, while no changes of total AKT expression were observed (Figure 6(b)). Furthermore, *β*-catenin–luciferase levels in total cell lysates was assayed by measuring luciferase activity. Knocking down PLOD1 in osteosarcoma cells significantly decreased *β*-catenin reporter luciferase activity (Figure 6(c)). Activation of the Wnt/*β*-catenin signaling pathway results in *β*-catenin translocation to the nucleus and transcription of Wnt target genes. According to the IF staining results, we found that inhibiting PLOD1 markedly reduced the translocation of *β*-catenin into the nucleus (Figure 6(d)). These results indicated that PLOD1 might regulate the *β*-catenin signal pathway.

## 4. Discussion

In this study, we found that PLOD1 was the only gene in the PLOD family that was elevated in osteosarcoma tissues when compared with nontumor tissues both in public datasets and in a validation cohort. Expression of PLOD1 in osteosarcoma tissues correlated with distance metastasis at diagnosis and advanced Enneking stage. Besides, we found that patients with a higher level of PLOD1 had worse prognosis than those with a lower level of PLOD1. PLOD2/3 expression had no significant association with tumor malignancy and clinical prognosis. These results gave us a hint that PLOD1 might be involved in the malignancy process of osteosarcoma. Moreover, we found that PLOD1 was mainly involved in the biological process of extracellular structure organization and promoted migration and invasion of MG-63 cells in vitro.

The PLOD family includes 3 members that are mainly responsible for lysyl hydroxylation, of which mutations have been shown to cause Ehlers-Danlos syndrome as well as Bruck syndrome type 2 [20], while the association between the PLOD family and cancer had rarely been studied. Expression of PLOD genes was observed to be elevated in clear cell renal cell carcinoma (ccRCC) when compared with normal kidney tissues, and genetic mutations of PLOD genes were presented in ~3% ccRCC tissues. Furthermore, they found that mRNA of PLOD genes was positively associated with the prognosis of ccRCC patients, while the expression pattern of PLOD genes in gastric cancer was different from those in ccRCC [14]. The expression of the PLOD family was also increased in gastric cancer compared with normal tissues. High PLOD1 and PLOD3 expression correlated with worse overall survival time in all GC, not diffuse-type GC patients, while the expression of PLOD2 in all GC has no significant association with overall survival time [16]. Therefore, it is common to see a specific gene play different roles in different types of cancers. In our study, PLOD1 expression, not PLOD2 and PLOD3 expression, increased in osteosarcoma tissues compared with normal tissues. And we found that PLOD1 was the only gene in the PLOD family that was significantly associated with the prognosis of osteosarcoma patients. Taken together, our study indicated that PLOD1 might be involved in the malignancy process of osteosarcoma.

In the present study, we found that PLOD1 was a potential biomarker in predicting the prognosis of osteosarcoma. PLOD1 was firstly used as a biochemical and histochemical marker for the identification and evaluation of bone mineral density [21]. With the development of genome and transcriptome sequencing technologies, genetic alteration of PLOD1 was found out to be closely related to various cancers. Analysis of PLOD1 expression in 177 colorectal cancer tissues revealed that patients in the high-PLOD1 group had a reduced recurrence-free survival compared with those in the low-PLOD1 group. Not only that, high-PLOD1 expression in gastric cancer is closely correlated with poor overall survival and progression-free survival, which strongly indicated that PLOD1 acted as a reliable prognostic biomarker in cancers [16].

A previous study had demonstrated that PLOD1 expression was regulated by hypoxia condition which was one of the main reasons that contributed to therapeutic resistance [22, 23]. Prostate cells were cultured under hypoxia, and the results of proteome showed that PLOD1 was one of the differentially expressed proteins that were involved with structural and binding processes [24]. Lower cross-link levels derived from PLODs most likely result in a faster degradation of collagen molecules, resulting in less ECM accumulation. The role of PLOD1 involved in the degradation of collagen and ECM construction might also play a crucial role in cancer metastasis. Another study found that miR-140-5p could directly bond with the 3′-UTR of PLOD1 and decreased the expression of PLOD1 in T24 and BOY cells. Small interfering RNA-mediated PLOD1 knockdown could dramatically inhibit migration and invasion of T24 cells [25]. These results were consistent with our findings. Using GO analysis, we found that PLOD1 might be involved in extracellular structure organization. Indeed, PLOD2/3 had been reported to be crucial for tumor invasion and metastasis. Inhibition of PLOD2 in breast cancer cells could obviously reduce the collagen accumulation and distance metastasis [26]. In this study, for the first time, we revealed that knockdown PLOD1 could inhibit proliferation, migration, and invasion of osteosarcoma cells. Expression of MMP-2 and vimentin was significantly decreased in si-PLOD1 MG-63 and U-2OS cells. Therefore, the present study may provide more reliable evidence that PLOD1 promoted in invasion of cancer cells.

The Wnt/*β*-catenin signaling pathway plays a key role in the biological behavior of osteosarcoma occurrence, differentiation, proliferation, and invasion [27, 28]. It is generally known that Wnt signaling is subdivided into canonical (*β*-catenin-dependent) and noncanonical (*β*-catenin-independent) pathways [29]. Therefore, the nuclear translocation of *β*-catenin is a prerequisite for the activation of the classical Wnt/*β*-catenin pathway. Nuclear translocation of *β*-catenin and its association with Tcf/Lef-1 factors can activate gene expression (mmp2, vimentin, and snail1) and cell proliferation in experimental systems [30]. In this study, we uncovered a novel gene, PLOD1, which could also be a regulator of the Wnt/*β*-catenin pathway. This gave us a hint that PLOD1 might be a potential therapeutic target in osteosarcoma.

## 5. Conclusion

PLOD1 was elevated in osteosarcoma and served as a prognostic biomarker. PLOD1 promoted proliferation, migration, and invasion of osteosarcoma cells by regulating the Wnt/*β*-catenin signaling pathway, which indicated PLOD1 might be a potential therapeutic target in osteosarcoma.

## Figures and Tables

**Table 1:** Univariate and multivariate Cox regression analyses of prognostic parameters in validation cohort.

	Univariate Cox regression		Multivariate Cox regression	
	HR (95% CI)	*P* value	HR (95% CI)	*P* value
Age (≥16 y vs. <16 y)	0.92 (0.93-1.01)	0.10	0.94 (0.87-1.02)	0.14
Gender (female vs. male)	2.01 (0.80-5.03)	0.14	—	—
Location (femur/tibia vs. other)	1.36 (0.42-4.34)	0.74	—	—
Lung metastasis at diagnosis (yes vs. no)	2.36 (1.28-7.50)	<0.01	1.69 (0.72-5.73)	0.02
Enneking stage (IIB-III vs. I-IIA)	3.32 (0.75-10.64)	<0.01	4.16 (1.34-12.90)	<0.01
PLOD1 (high vs. low)	1.20 (0.99-1.51)	<0.01	1.23 (1.02-1.47)	0.03
PLOD2 (high vs. low)	1.11 (0.92-1.34)	0.28	—	—
PLOD3 (high vs. low)	0.89 (0.63-1.20)	0.45	—	—

## Data Availability

The authors are responsible for the submission and agree to the open data policy.
